# Smart investment of virus RNA testing resources to enhance Covid-19 mitigation

**DOI:** 10.1371/journal.pone.0259018

**Published:** 2021-11-30

**Authors:** Hossein Gorji, Markus Arnoldini, David F. Jenny, Wolf-Dietrich Hardt, Patrick Jenny

**Affiliations:** 1 Laboratory of Multiscale Studies in Building Physics, Empa, Dübendorf, Switzerland; 2 Department of Health Sciences and Technology, Swiss Federal Institute of Technology, Zürich, Switzerland; 3 Department of Mathematics, Swiss Federal Institute of Technology, Zürich, Switzerland; 4 Institute of Microbiology, D-BIOL, Swiss Federal Institute of Technology, Zürich, Switzerland; 5 Department of Mechanical and Process Engineering, Swiss Federal Institute of Technology, IFD, Zürich, Switzerland; University of Illinois College of Veterinary Medicine, UNITED STATES

## Abstract

A variety of mitigation strategies have been employed against the Covid-19 pandemic. Social distancing is still one of the main methods to reduce spread, but it entails a high toll on personal freedom and economic life. Alternative mitigation strategies that do not come with the same problems but are effective at preventing disease spread are therefore needed. Repetitive mass-testing using PCR assays for viral RNA has been suggested, but as a stand-alone strategy this would be prohibitively resource intensive. Here, we suggest a strategy that aims at targeting the limited resources available for viral RNA testing to subgroups that are more likely than the average population to yield a positive test result. Importantly, these pre-selected subgroups include symptom-free people. By testing everyone in these subgroups, in addition to symptomatic cases, large fractions of pre- and asymptomatic people can be identified, which is only possible by testing-based mitigation. We call this strategy smart testing (ST). In principle, pre-selected subgroups can be found in different ways, but for the purpose of this study we analyze a pre-selection procedure based on cheap and fast virus antigen tests. We quantify the potential reduction of the epidemic reproduction number by such a two-stage ST strategy. In addition to a scenario where such a strategy is available to the whole population, we analyze local applications, e.g. in a country, company, or school, where the tested subgroups are also in exchange with the untested population. Our results suggest that a two-stage ST strategy can be effective to curb pandemic spread, at costs that are clearly outweighed by the economic benefit. It is technically and logistically feasible to employ such a strategy, and our model predicts that it is even effective when applied only within local groups. We therefore recommend adding two-stage ST to the portfolio of available mitigation strategies, which allow easing social distancing measures without compromising public health.

## Introduction

The Covid-19 pandemic has evaded containment measures, both during the initial stage and the subsequent waves of infections. Public health responses have therefore shifted towards mitigating its effects. Safe and effective vaccines have been developed and are now produced at impressive speed, and vaccination programs are progressing fast in many countries. But supply of vaccines as well as of reliable tests to detect viral RNA are still costly and resource-intensive, and many countries still have to rely on a portfolio of mitigation strategies to keep the SARS-CoV-2 virus from spreading. These include hygiene measures, social distancing (including temporary lock-down of large parts of the economy), testing for virus infections and contact tracing. This combination of measures reduces the burden on the healthcare system by easing the demand for intensive care. Even with ongoing vaccination, mitigation measures are still important: with increasing fractions of the population being vaccinated, curbing virus spread helps reduce selection for virus mutants that can evade the protection provided by vaccines and protect when immunity wanes in vaccinated people over time [1]. The current portfolio of mitigation strategies has two major shortcomings. First, it leaves many infected people with mild or no symptoms undetected [2], and therefore renders them more likely to infect others. Second, as social distancing measures limit business that is considered non-essential, it imposes a severe economic toll, with additional consequences for society and public health. We therefore need to consider alternative mitigation strategies that allow gradual re-opening of the economy without a surge in Covid-19 cases, until herd immunity via vaccination or convalescence from infection can be reached.

Detecting infected people without symptoms is especially important, as failing to do so limits the effect of contact tracing and thus makes it impossible to break infection chains. Between 11.5% and 43.2% of all infected cases are estimated to develop no symptoms [3–5], and asymptomatic people have been estimated to be between 10% and 100% as infectious as symptomatic ones (based on viral load) [3, 6, 7]. Although some asymptomatic infections can be found by the contact tracing, the performance is limited as the scheme is focused on contacts of symptomatic individuals. In fact, we have recently shown analytically that contact tracing alone is insufficient to reduce *R*_*eff*_ to 1 even for the most optimistic assumptions, which is largely due to the asymptomatic cases [8]; contact tracing can still be a valuable mitigation tool, but it needs to be complemented by other measures.

Recently, we and others have analyzed how mass-testing random samples of the population and quarantine of positive cases could work to mitigate the pandemic [8–11]. Importantly, by testing for virus RNA, a marker for active infections, also asymptomatic but infectious people can be detected. We estimated how many tests would be needed per day do mitigate the pandemic, i.e. to reduce the basic reproduction number *R*_0_ of the pandemic from 2.4–3 (for the wild SARS-CoV-2 [12]) to an effective reproduction number of *R*_*eff*_ = 1. Our model shows that this is possible, but that unselective mass-testing would incur a huge strain on testing resources and infrastructure.

In this paper, we analyze an option to target the available numbers of virus RNA tests to pre-selected sub-populations, a strategy we term smart-testing (ST). We first analyze the mitigating power of ST in general, and then a specific option to identify sub-populations by pre-screening with quick and cheap virus antigen tests. This two-stage testing approach has recently been proposed [13], but has not been analyzed quantitatively. Using mathematical modeling, we show that such a two-stage ST strategy greatly reduces the required number of virus RNA tests and increases the mitigation power of testing, even if applied only locally (e.g. in a company or school).

## Materials and methods

### Basic model

**Fig 1:**
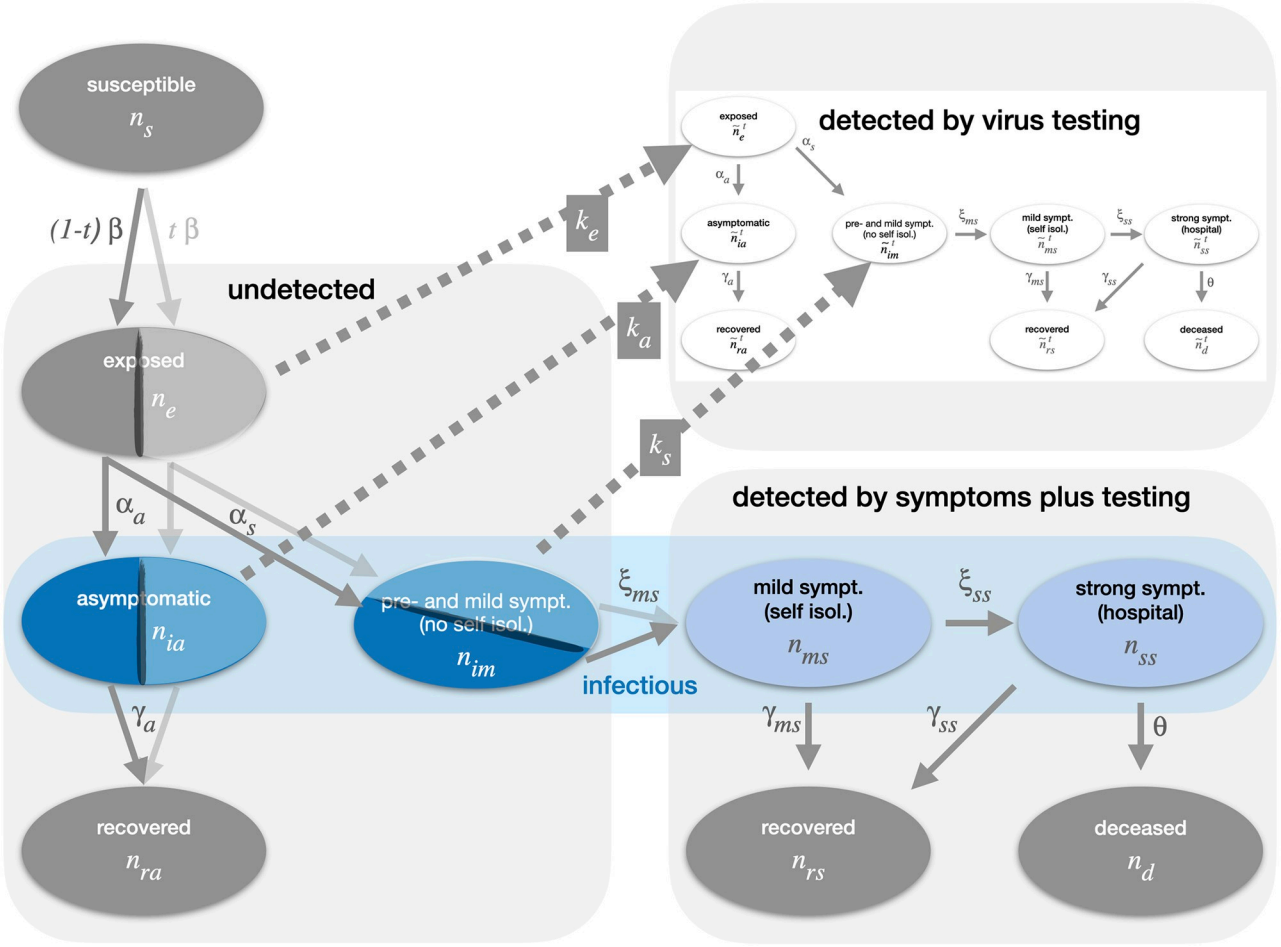
Graphical illustration of the modeling approach showing the dependencies within the system describing the dynamics of the susceptible, undetected and detected infected populations. It is crucial that the model distinguishes between individuals detected by symptoms (light blue), and those detected by virus testing (inserted graph). The detection rates of exposed, asymptomatic and mild symptomatic persons due to testing are proportional to *k*_*e*_, *k*_*a*_ and *k*_*s*_, respectively. These individuals are then accounted for in the inserted graph with the white compartments, which is very similar as the main one, except that there is no node for susceptible persons (since by definition a susceptible person cannot be detected infected) and that there exist sources due to testing (dotted arrows) instead of sinks.

We extended a generalized SEIR model [14–18] we have previously developed [9]. It specifically accounts for detected and undetected infected people. Detection can happen via symptoms or via testing. This is important, as pre-symptomatic and asymptomatic virus carriers play a central role in virus transmission [2, 19, 20]. In addition, the model allows for the study of cross-infection, i.e. the inflow of infections into the modeled population from an external source, offering the opportunity to study mitigation scenarios localized to sub-populations such as cities, companies, or schools. A visualization of the modeling approach is shown in Fig 1. The variables *n*_*s*_, *n*_*e*_ and *n*_*ia*_ denote the numbers of susceptible, exposed and asymptomatic persons, respectively. Further, *n*_*im*_ is the number of pre- or mild symptomatic people (not in isolation), *n*_*ms*_ that of mild symptomatic people (in isolation), *n*_*ss*_ that of hospitalized patients with strong symptoms, and *n*_*ra*_, *n*_*rs*_ and *n*_*d*_ are the numbers of asymptomatic recoveries, symptomatic recoveries and deaths, respectively. Note that the compartments of exposed (subscript *e*), asymptomatic (subscript *ia*) and mild symptomatic (subscript *im*) are split into sub-compartments representing the fractions *t* and (1 − *t*) which do (superscript *t*) and do not (superscript *nt*) participate in repetitive testing, respectively. Table 1 provides a description of the employed symbols.

**Table 1:** Terminology and nomenclature of model parameters and variables.

terminology	meaning
susceptible	persons of the considered population who are susceptible and thus can potentially get infected
exposed	infected persons; can not yet transmit the virus
asymptomatic	infected persons without symptoms; can transmit the virus
pre- and mild sympt. (no self isol.)	infected persons with no or mild symptoms; infectious, but not isolated
mild sympt. (self isol.)	infected persons with mild symptoms; infectious and isolated
strong symptomatic	infected persons with strong symptoms and thus hospitalized; isolated
deceased	persons who died
recovered	persons who recovered
detected	isolated either after positive testing or after falling ill
undetected	persons who are either exposed, asymptomatic or mild symptomatic, but were never contained
transmissive	persons who are either asymptomatic or symptomatic
mild social distancing	$R_{\text{eff}}=1.6$ , if the infection rate is reduced by 33% via social distancing
**variables**	
*n*_*s*_ and $n_{s}^{0}$	numbers of susceptible and initially susceptible persons, respectively
$n_{e}^{t}$ , $n_{e}^{nt}$, ${\tilde{n}}_{e}$ and $n_{e}^{tot}$	numbers of exposed persons; not tested, tested and in total, respectively
$n_{ia}^{t}$ , $n_{ia}^{nt}$, ${\tilde{n}}_{ia}$, $n_{ia}^{tot}$ and $n_{ia}^{e}$	numbers of asymptomatic persons; not tested, tested, in total and in external population, respectively
$n_{im}^{t}$ , $n_{im}^{nt}$, ${\tilde{n}}_{im}$, $n_{im}^{tot}$ and $n_{im}^{e}$	numbers of persons with mild symptoms during first day; not tested, tested, in total and in ext. population, resp.
*n*_*ms*_, ${\tilde{n}}_{ms}$, $n_{ms}^{tot}$ and $n_{ms}^{e}$	numbers of persons with mild symptoms after first day; not tested, tested, in total and in external population, respectively
*n*_*ss*_, ${\tilde{n}}_{ss}$ and $n_{ss}^{tot}$	numbers of persons with strong symptoms; not tested, tested and in total, respectively
*n*_*ra*_, ${\tilde{n}}_{ra}$ and $n_{ra}^{tot}$	numbers of recovered persons who had no symptoms; not tested, tested and in total, respectively
*n*_*rs*_, ${\tilde{n}}_{rs}$ and $n_{rs}^{tot}$	numbers of recovered or deceased persons who had symptoms; not tested, tested and in total, respectively
$n_{i}^{undet}$	undetected infected persons: *n*_*ie*_+ *n*_*ia*_+ *n*_*im*_
$n_{i}^{det}$	detected infected persons: $n_{ms}+n_{ss}+{\tilde{n}}_{ms}+{\tilde{n}}_{ss}+{\tilde{n}}_{ie}+{\tilde{n}}_{ia}+{\tilde{n}}_{im}$
**parameters**	
*β* and $\tilde{\beta }$	rate coefficients for infection without and with cross-infections
*t*	fraction participating in repetitive testing
*ϵ*	ratio between infection rate of self-isolated and non-quarantined symptomatic cases
*α*_*a*_ and *α*_*s*_	rate coefficients for latency of asymptomatic and symptomatic cases, respectively
*γ*_*a*_, *γ*_*ms*_ and *γ*_*ss*_	rate coefficients for recovery
*ξ*_*ms*_ and *ξ*_*ss*_	rate coefficients for successively stronger symptoms
*k*_*e*_, *k*_*a*_ and *k*_*s*_	rate coefficients accounting for testing
*r*_*ec*_	ratio of external to overall contacts
*p*^*e*^ and *p*	prevalence of external and main population, respectively
*p*^*h*^, *p*^*l*^ and *r*_*p*_	prevalence of high-prevalence sub-population, low-prevalence complement and their ratio, respectively
$R_{0}$	basic reproduction number without mitigation
$R_{\text{eff}}$	effective reproduction number with mitigation
$R_{\text{eff}}^{wt}$	effective reproduction number subject to testing
*N*	testing interval
*η*	fraction of virus-RNA false negative test results
*f*_*n*_ and *f*_*r*_	fraction of antigen false negative and false positive test results, respectively
*r*_*mt*_, *r*_*rna*_ and *r*_*ag*_	fraction of population subject to mass-testing, RNA testing and antigen testing per day, respectively
$r_{s_{1}}$ , $r_{s_{2}}$ and *r*_*s*_	fraction of antigen tested population with positive, negative and either results, respectively
**operators & functions**	
E(·)	expectation
$P_{A}$ and Prob{A}	probability of the event A
*σ*(*η*, *τ*_*proc*_, *r*_*mt*_) and *r*_*wt*_	reduction in the reproduction number due to the mass-testing without and with cross-infections, respectively

The described mechanism can then be cast into a set of ordinary differential equations, which governs the dynamics of the population in each compartment. Without accounting for infections via external contacts the system reads

$$\begin{array}{ccc} {\dot{n}}_{s} & = & -\beta \left(n_{ia}/2+n_{im}+\epsilon n_{ms}\right)\frac{n_{s}}{n_{s}^{0}}, \end{array}$$ (1)

$$\begin{array}{ccc} {\dot{n}}_{e}^{t} & = & t\beta \left(n_{ia}/2+n_{im}+\epsilon n_{ms}\right)\frac{n_{s}}{n_{s}^{0}}\;-\;\left(\alpha_{a}+\alpha_{s}\right)n_{e}^{t}\;-k_{e}n_{e}^{t}, \end{array}$$ (2)

$$\begin{array}{ccc} {\dot{n}}_{e}^{nt} & = & \left(1-t\right)\;\beta \left(n_{ia}/2+n_{im}+\epsilon n_{ms}\right)\frac{n_{s}}{n_{s}^{0}}\;-\;\left(\alpha_{a}+\alpha_{s}\right)n_{e}^{nt}, \end{array}$$ (3)

$$\begin{array}{ccc} {\dot{n}}_{ia}^{t} & = & \alpha_{a}n_{e}^{t}\;-\;\gamma_{a}n_{ia}^{t}\;-\;k_{a}n_{ia}^{t}, \end{array}$$ (4)

$$\begin{array}{ccc} {\dot{n}}_{ia}^{nt} & = & \alpha_{a}n_{e}^{nt}\;-\;\gamma_{a}n_{ia}^{nt}, \end{array}$$ (5)

$$\begin{array}{ccc} {\dot{n}}_{im}^{t} & = & \alpha_{s}n_{e}^{t}\;-\;\xi_{ms}n_{im}^{t}\;-\;k_{s}n_{im}^{t}, \end{array}$$ (6)

$$\begin{array}{ccc} {\dot{n}}_{im}^{nt} & = & \alpha_{s}n_{e}^{nt}\;-\;\xi_{ms}n_{im}^{nt}, \end{array}$$ (7)

$$\begin{array}{ccc} {\dot{n}}_{ra} & = & \gamma_{a}n_{ia}, \end{array}$$ (8)

$$\begin{array}{ccc} {\dot{n}}_{ms} & = & \xi_{ms}n_{im}\;-\;\left(\gamma_{ms}+\xi_{ss}\right)n_{ms}, \end{array}$$ (9)

$$\begin{array}{ccc} {\dot{n}}_{ss} & = & \xi_{ss}n_{ms}\;-\;\gamma_{ss}n_{ss}\;\;\;\;\;\text{and} \end{array}$$ (10)

$$\begin{array}{ccc} {\dot{n}}_{rs} & = & \gamma_{ss}n_{ss}\;+\;\gamma_{ms}n_{ms}. \end{array}$$ (11)

Note that $n_{e}=n_{e}^{nt}+n_{e}^{t}$, $n_{ia}=n_{ia}^{nt}+n_{ia}^{t}$ and $n_{im}=n_{im}^{nt}+n_{im}^{t}$.

### Cross-infections

To account for the cross-infection between internal and external populations, a key parameter is the ratio of external to overall contacts *r*_*ec*_ (see e.g. [21] for more advanced cross-infection models). To keep the epidemic model in an explicit form and to avoid introducing further new parameters, we apply a simplifying assumption of a constant prevalence ratio between external and internal populations, for all infectious compartments. Then, it is straight-forward to show that the cross-infections become a modification

$$\begin{array}{c} \tilde{\beta }=\beta (1+r_{ec}(\frac{p^{e}}{p}-1)) \end{array}$$ (12)

of the infection rate *β*, where *p*^*e*^ and *p* are the prevalence of external and internal populations, respectively.

The $R_{0}$ with no testing can then be described in the linear regime as follows

$$\begin{array}{ccc} R_{0} & = & \frac{\tilde{\beta }\alpha_{s}}{\alpha_{a}+\alpha_{s}}[\frac{\alpha_{a}}{2\gamma_{a}\alpha_{s}}+\frac{1}{\xi_{ms}}+\frac{\epsilon }{\gamma_{ms}+\xi_{ss}}]. \end{array}$$ (13)

### Testing

During a testing campaign which invites a number of people to be tested at regular intervals and quarantines positive cases, people from exposed, asymptomatic and pre/mild-symptomatic compartments are removed by the rates *k*_*e*_, *k*_*a*_ and *k*_*s*_, respectively. The dynamics of the individuals identified through the testing then follows

$$\begin{array}{ccc} {\dot{\tilde{n}}}_{e}^{t} & = & -\left(\alpha_{a}+\alpha_{s}\right){\tilde{n}}_{e}^{t}\;+\;k_{e}n_{e}^{t}, \end{array}$$ (14)

$$\begin{array}{ccc} {\dot{\tilde{n}}}_{ia}^{t} & = & \alpha_{a}{\tilde{n}}_{e}^{t}\;-\;\gamma_{a}{\tilde{n}}_{ia}^{t}\;+\;k_{a}n_{ia}^{t}, \end{array}$$ (15)

$$\begin{array}{ccc} {\dot{\tilde{n}}}_{im}^{t} & = & \alpha_{s}{\tilde{n}}_{e}^{t}\;-\;\xi_{ms}{\tilde{n}}_{im}^{t}\;+\;k_{s}n_{im}^{t}, \end{array}$$ (16)

$$\begin{array}{ccc} {\dot{\tilde{n}}}_{ra}^{t} & = & \gamma_{a}{\tilde{n}}_{ia}^{t}, \end{array}$$ (17)

$$\begin{array}{ccc} {\dot{\tilde{n}}}_{ms}^{t} & = & \xi_{ms}{\tilde{n}}_{im}^{t}\;-\;\left(\gamma_{ms}+\xi_{ss}\right){\tilde{n}}_{ms}^{t}, \end{array}$$ (18)

$$\begin{array}{ccc} {\dot{\tilde{n}}}_{ss}^{t} & = & \xi_{ss}{\tilde{n}}_{ms}^{t}\;-\;\gamma_{ss}{\tilde{n}}_{ss}^{t}\;\;\;\;\;\text{and} \end{array}$$ (19)

$$\begin{array}{ccc} {\dot{\tilde{n}}}_{rs}^{t} & = & \gamma_{ss}{\tilde{n}}_{ss}^{t}\;+\;\gamma_{ms}{\tilde{n}}_{ms}^{t}. \end{array}$$ (20)

The detection rates *k*_*e*_, *k*_*a*_, *k*_*s*_ are computed analogous to [9], and they crucially depend on the number of people tested at regular intervals, and the length of these intervals. Using the same approach as when deriving Eq (13), the effective reproduction number with testing can be computed based on the analytical formula (valid in the linear regime)

$$\begin{array}{ccc} R_{\text{eff}}^{wt,all} & = & \;\frac{\tilde{\beta }\alpha_{s}}{\alpha_{a}+\alpha_{s}+k_{e}}[\frac{\alpha_{a}}{2\left(\gamma_{a}+k_{a}\right)\alpha_{s}}+\frac{1}{\xi_{ms}+k_{s}}(1+\frac{\epsilon \xi_{ms}}{\gamma_{ms}+\xi_{ss}})], \end{array}$$ (21)

and if only a fraction *t* participates,

$$\begin{array}{ccc} R_{\text{eff}}^{wt} & = & t\;R_{\text{eff}}^{wt,all}\;+\;\left(1-t\right)\;R_{0}. \end{array}$$ (22)

Based on Eq (22), the number of required tests per day can be estimated for a desired reduction in the reproduction number (reproduction number reduction factor), i.e., $R_{\text{eff}}^{wt}/R_{0}$, as a function of test characteristics and cross-infections (as shown below).

The sensitivity of $R_{\text{eff}}^{wt,all}/R_{0}$ on fraction, infectiousness and recovery time of asymptomatic persons, and on infectiousness of isolated cases has been investigated previously and is discussed in [9].

### Two-stage smart testing

Suppose *p*^*h*^ is the probability of finding an infected individual in a pre-selected sub-population, whereas *p*^*l*^ is the overall prevalence. An ST strategy targets available tests at this sub-population. The number of tests required to achieve the same effective reproduction number then reduces by the factor

$$\begin{array}{ccc} r_{p} & = & \frac{p^{h}}{p^{l}}; \end{array}$$ (23)

see [22] for details. One approach to identify a sub-population with high positive predictive value is to deploy rapid antigen tests [23], which are less sensitive and less specific than virus RNA tests, but can still be used for pre-testing. Let’s assume that these antigen tests have sensitivity and specificity of *S*_*e*_ and *S*_*p*_, respectively. Antigen mass-testing is performed in a sub-population that has prevalence *p*^*l*^. The sub-population identified by a positive antigen test result is composed of a fraction

$$\begin{array}{ccc} r_{s_{1}} & = & S_{e}p^{l} \end{array}$$ (24)

of positive cases and a fraction

$$\begin{array}{ccc} r_{s_{2}} & = & \left(1-S_{p}\right)\left(1-p^{l}\right) \end{array}$$ (25)

of negative cases. Therefore, the screened fraction of the sub-population is

$$\begin{array}{ccc} r_{s} & = & r_{s_{1}}+r_{s_{2}}=p^{l}S_{e}+\left(1-S_{p}\right)\left(1-p^{l}\right), \end{array}$$ (26)

and the corresponding positive predictive value is

$$\begin{array}{ccc} p^{h} & = & \frac{S_{e}p^{l}}{r_{s}}, \end{array}$$ (27)

leading to the ratio of the positive predictive value to the prevalence

$$\begin{array}{ccc} r_{p} & = & \frac{S_{e}}{r_{s}}. \end{array}$$ (28)

Now, if we apply the virus RNA mass-testing strategy on the pre-screened sub-population, we achieve the same mitigation impact as without pre-screening, but with *r*_*p*_ times fewer RNA tests. Consider a scenario where we invite a random fraction *r*_*ag*_ of the population per day for antigen testing. A fraction *r*_*s*_ of this population will be pre-screened based on the positive antigen test results. Therefore, compared to the original population, the fraction of the pre-screened sub-population is *r*_*ag*_*r*_*s*_. This fraction should match the number of required RNA tests relative to the overall population, i.e., *r*_*mt*_/*r*_*p*_, leading to the fraction

$$\begin{array}{c} r_{ag}=\frac{r_{mt}}{S_{e}} \end{array}$$ (29)

of the susceptible population to be tested daily by antigen tests, and the fraction

$$\begin{array}{c} r_{rna}=\frac{r_{mt}}{r_{p}}=r_{mt}(p^{l}+\frac{\left(1-p^{l}\right)\left(1-S_{p}\right)}{S_{e}}) \end{array}$$ (30)

of the susceptible population to be tested daily by RNA tests. Concrete examples of how this strategy would work are discussed in S1 File.

### Parameter estimation

Our model is closed once the rate coefficients are estimated. We estimate their values from published data, mainly from [4, 24]. The process of parameter estimation is described in detail in [9]. The listed values in Table 2 are used for all analyses if not explicitly stated otherwise. Note that these values can easily be adapted, if more reliable data becomes available or to adapt for new virus variants. It is to be emphasized that our aim is not to perform high-fidelity scenario predictions; we are interested in using the model to explore different mitigation strategies, while at the same time taking the parameters from a realistic range.

**Table 2:** List of estimated parameters and initial values. Note that our model allows to easily replace any of these parameters by more precise estimates, as more data become available. The initial values of all numbers except *n*_*e*_ are set to zero.

**parameters**	**value**
*β*	0.670 (1/day)
*ϵ*	0.1
*α*_*a*_	0.078 (1/day)
*α*_*s*_	0.156 (1/day)
*γ*_*a*_	0.087 (1/day)
*ξ*_*ms*_	0.667 (1/day)
*γ*_*ms*_	0.08 (1/day)
*ξ*_*ss*_	0.02 (1/day)
*γ*_*ss*_	0.091 (1/day)
**initial condition**	**value**
$n_{e}\left(0\right)/n_{s}^{0}$	1.5663e-07

For *k*_*e*_, *k*_*a*_ and *k*_*s*_ polynomial fits as functions of the test frequency *ν* = *N*^−1^, the test processing time *τ*_proc_ and the sensitivity *S*_*e*_ were derived based using the Monte Carlo scheme proposed in [9]. The numerical simulations are performed to estimate the map

$$\begin{array}{ccc} k_{e,a,s} & = & S_{e}\frac{\nu }{\text{max}(\nu ,0.02)}\sum_{i=0}^{8}\{\sum_{j=0}^{8}a_{ij}^{e,a,s}\text{max}{\left(\nu ,0.02\right)}^{i}\tau_{\text{proc}}^{j}\} \end{array}$$ (31)

with the coefficients listed in Tables 3–5. The sensitivity of $R_{\text{eff}}^{wt,all}/R_{0}$ on fraction, infectiousness and recovery time of asymptomatic persons, and on infectiousness of isolated cases has been investigated previously and is discussed in [9], the results lie in the 20% margin of the base scenario estimate. Our results are comparable to other theoretical studies on repetitive testing interventions. For example in [10], Grassly et al estimate that weekly PCR testing of health-care workers and other high-risk groups reduce their contribution to the reproductive number by 23%. This is in agreement with lower range of our estimates.

**Table 3:** Coefficients for *k*_*e*_.

$a_{ij}^{e}$	*j* = 0	*j* = 1	*j* = 2	*j* = 3	*j* = 4
*i* = 0	0.018539229336842107	-0.09476767971578948	0.23754212988421056	-0.31898996134736846	0.24946536628421057
*i* = 1	3.3429447546	-3.646627282989474	3.147180452073684	-3.2481430706421053	3.4357770562105268
*i* = 2	40.685408791147374	-182.88693803556845	442.6709494575684	-593.3466887325369	471.5638445329369
*i* = 3	-292.94616634534736	1474.0521871396422	-3692.3787475579265	5063.631152512653	-4106.345496458273
*i* = 4	1159.2408378588423	-6479.211915480927	16567.416520828505	-23001.232923326268	18858.58702937443
*i* = 5	-2599.890797717326	15533.14406514799	-40315.54022801347	56446.88233697165	-46592.829024039675
*i* = 6	3351.760631076726	-20878.951865821622	54777.04604047197	-77179.31027068506	64024.25577984621
*i* = 7	-2300.1098995631683	14718.085976431023	-38933.76091912583	55143.346642427634	-45941.81570369866
*i* = 8	655.6309671533791	-4272.828227769979	11381.117283974212	-16197.732902293475	13552.953997245957
	*j* = 5	*j* = 6	*j* = 7	*j* = 8	
*i* = 0	-0.11754100905263158	0.03290260227368422	-0.0050406110315789465	0.0003253794315789474	
*i* = 1	-2.435861701663158	0.9880725905894738	-0.20850276472631582	0.017792254789473685	
*i* = 2	-228.37560154293686	66.18621633406316	-10.547550353021053	0.7106474302210527	
*i* = 3	2025.512421460137	-596.9339981232	96.60795029860002	-6.603876381673684	
*i* = 4	-9394.811885514831	2793.3230394198317	-455.68673762265263	31.378403478821053	
*i* = 5	23341.69862205579	-6972.93556127461	1142.141580256737	-78.92937777256843	
*i* = 6	-32210.995099848973	9658.587386715253	-1587.414074715642	110.0469483514316	
*i* = 7	23203.814193624534	-6983.3054771973675	1151.7848650712106	-80.1231082192842	
*i* = 8	-6873.6393915756535	2077.235031249063	-344.03805843328416	24.03348617569474	

**Table 4:** Coefficients for *k*_*a*_.

$a_{ij}^{a}$	*j* = 0	*j* = 1	*j* = 2	*j* = 3	*j* = 4
*i* = 0	-0.013338239585263159	-0.0037355772863157896	0.012284835485263159	-0.023525432102105264	0.026599042395789477
*i* = 1	2.7283052407778947	0.03963067792315789	-0.485180567471579	0.9189839789189473	-1.1235567501252632
*i* = 2	-34.7847201298421	3.2288619514231582	6.3584869198305265	-12.932267301445263	16.97368976401053
*i* = 3	188.24729323945897	-30.968278367657895	-42.44977271575158	95.14314633116105	-128.77327005771897
*i* = 4	-549.662511683221	117.42120811947893	156.96397743496001	-374.05940497267056	512.6100682210453
*i* = 5	932.8904259660537	-231.71187123097894	-328.2568437220274	808.4191246539842	-1114.397633556318
*i* = 6	-921.2322593889927	252.4468853533895	384.55357319074426	-963.7091607163159	1332.8790570065862
*i* = 7	491.10718918996105	-144.3079834166484	-235.01576097575267	594.6193663146779	-824.1791302646548
*i* = 8	-109.2823545153516	33.85214562213474	58.341137559020005	-148.42181900675052	206.0342125118958
	*j* = 5	*j* = 6	*j* = 7	*j* = 8	
*i* = 0	-0.017045760490526318	0.006156749685263157	-0.0011733398389473684	9.196158000000001e-05	
*i* = 1	0.7673923141810527	-0.28875596389789476	0.05641228811684211	-0.004486161666315789	
*i* = 2	-12.254629729042106	4.7927744802515795	-0.9627632359178948	0.0781914063894737	
*i* = 3	95.36183359285475	-38.08544252607789	7.781309954608421	-0.640652335943158	
*i* = 4	-384.1950667986979	155.28661921667788	-32.06493852418842	2.663484104248421	
*i* = 5	841.5772778162421	-343.09262817761686	71.4160458255579	-5.973265647590527	
*i* = 6	-1012.3438742142381	415.56314118046106	-87.07242619780212	7.324722206767368	
*i* = 7	628.9057400434548	-259.6481263516411	54.705063960154746	-4.624294434685263	
*i* = 8	-157.83329183060212	65.47830378097895	-13.860063737797894	1.1764327101673686	

**Table 5:** Coefficients for *k*_*s*_.

$a_{ij}^{s}$	*j* = 0	*j* = 1	*j* = 2	*j* = 3	*j* = 4
*i* = 0	-0.0009963538557894737	0.0030426290842105264	0.002929494309473684	-0.012624825694736844	0.017003742078947368
*i* = 1	0.19370539852947372	-0.17021331099052633	-0.4265393606652632	1.4240815158389475	-1.827376739754737
*i* = 2	2.748562720728421	2.943314836131579	4.802588333093684	-20.928215446226318	28.38100506232
*i* = 3	-37.556618219421054	-14.906728585842107	-18.698430300345265	124.32328115307054	-181.43119588679053
*i* = 4	162.14758755986105	32.92481612183263	26.70939806833053	-374.3822961690537	593.8607761656137
*i* = 5	-346.3433266714021	-33.40562806400526	10.719477009116842	614.1822640775348	-1070.9305922709148
*i* = 6	398.87021852355264	10.119148786016842	-70.60187661842106	-552.3179621382201	1074.7638607438191
*i* = 7	-237.5294886148926	6.483619998571579	69.53752105108948	254.59325427336842	-563.9975311189022
*i* = 8	57.471931823320006	-3.98267311462	-22.098033786276844	-46.75505554074421	121.0041209092421
	*j* = 5	*j* = 6	*j* = 7	*j* = 8	
*i* = 0	-0.012246990108421053	0.0048864812547368425	-0.0010106009178947367	8.434615578947368e-05	
*i* = 1	1.281645008369474	-0.5039822183347369	0.10354873625578948	-0.008632244607368422	
*i* = 2	-20.470568547351583	8.20588356083579	-1.711361484131579	0.1444130603210526	
*i* = 3	135.69176203718317	-55.757562681462105	11.851315264812632	-1.0154049050978948	
*i* = 4	-461.9595691537526	194.71959026669475	-42.17342497043053	3.667159603817895	
*i* = 5	869.0581741751727	-376.13346945625375	83.02429993771686	-7.325352842474737	
*i* = 6	-912.0883403592874	405.5672493333842	-91.2276132631758	8.16465164359579	
*i* = 7	501.4194515758738	-229.04311115230527	52.47483163637158	-4.76074156747579	
*i* = 8	-112.80829513671789	52.89780975515369	-12.332257236845264	1.133170418616842	

## Results

### Smart testing as a mitigation strategy

**Fig 2:**
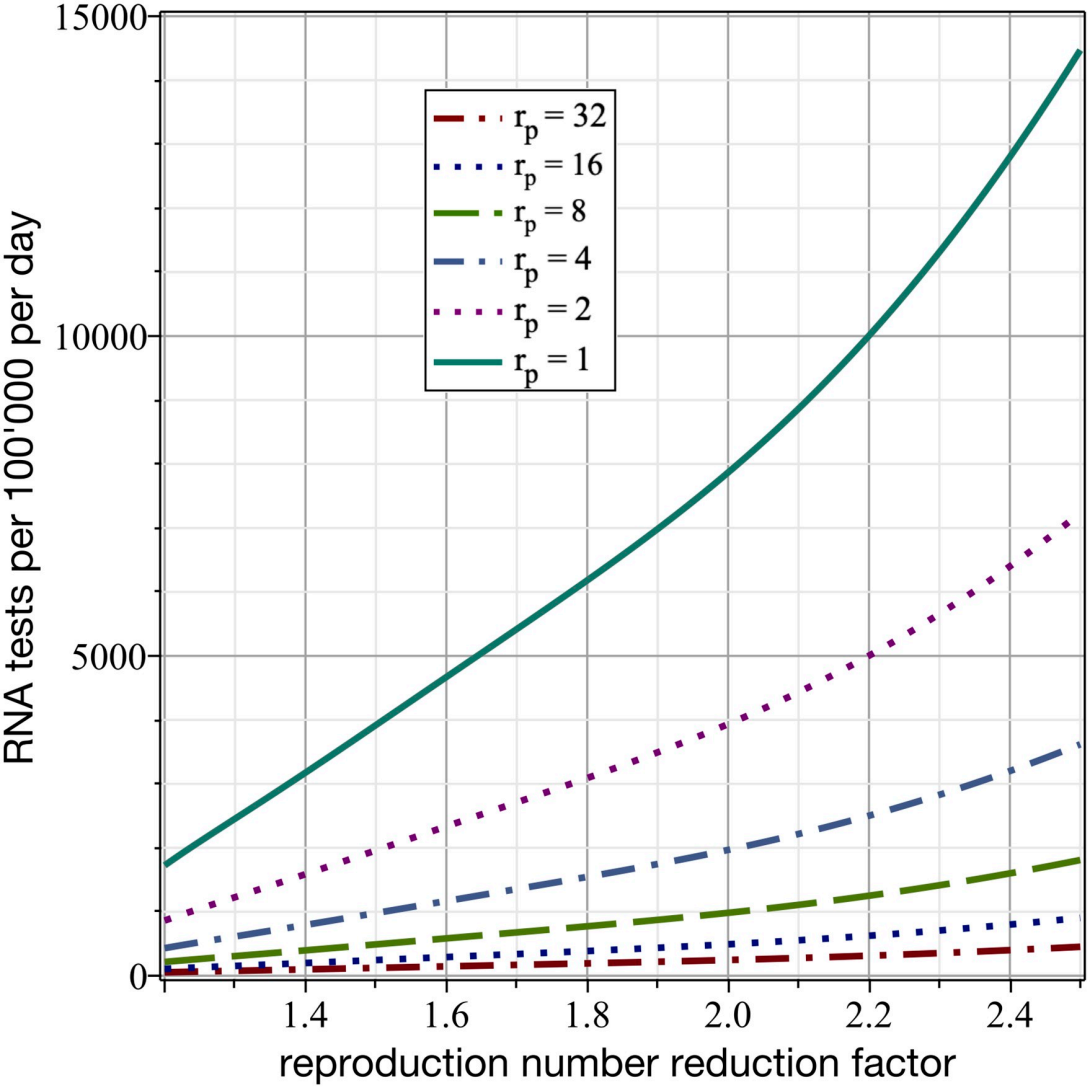
Numbers of required virus RNA tests per 100’000 people per day for ST to achieve a specified reproduction number reduction factor depending on the ratio of positive predictive value to the overall population prevalence. A 5% false negative rate and one day delay is assumed for the virus RNA tests.

An ST strategy relies on the targeting of available virus RNA tests to anti-gen test positive population. Importantly, such a strategy dramatically reduces the number of tests needed relative to random testing of the overall population to identify the same number of infected people. If these people are then quarantined, a mitigating effect is achieved. For this effect to be strong enough to achieve *R*_*eff*_ = 1, the ratio of the positive predictive value to the prevalence needs to be sufficiently high. In fact, the factor by which the number of required virus RNA tests can be reduced is equal to that ratio. Fig 2 shows the number of required virus RNA tests per 100’000 people per day to achieve a specified reproduction number reduction factor for different values of this ratio. For example, if the tested subpopulation has a positive predictive value 32 times higher than the prevalence, then only 246 virus RNA tests per 100’000 people per day would be required to reduce the reproduction number by a factor of two (a 95% sensitivity and one day delay is assumed here for virus RNA tests). These numbers indicate that ST can be a viable mitigation strategy, as the number of tests needed to achieve a sufficient reduction in *R*_*eff*_ is already available in several countries.

Here, we investigate the ST approach based on a pre-selection by virus antigen tests. Depending on the sensitivity and specificity of the antigen test (*S*_*e*_ and *S*_*p*_, respectively), we can calculate the fraction of the population which gets pre-selected in the first round of tests as *r*_*s*_ = *pS*_*e*_ + (1 − *p*)(1 − *S*_*p*_), where *p* is the prevalence in the population. The factor by which the probability of identifying an infected person increases in the antigen test positive population is then *r*_*p*_ = *S*_*e*_/*r*_*s*_. For example, with the realistic values of *S*_*e*_ = 70% and *S*_*p*_ = 99%, and with *p* = 0.3%, one obtains *r*_*s*_ = 0.012 and *r*_*p*_ = 58. Both values increase for lower false negative and false positive rates. Further, the ratio decreases for a higher *p*, which highlights the importance of starting with such a mitigation measure as early as possible in the epidemic. In the example considered here, 136 virus RNA tests per 100’000 people per day (a processing time of one day and a sensitivity of 95% for the virus RNA tests are assumed) would be required to reduce the reproduction number by a factor of two. Applying these 136 virus RNA tests to people from the antigen test positive population has the same effect as virus RNA-testing 7’870 random persons without pre-selection. This dramatically smaller number of required virus RNA tests (*r*_*p*_ = 58 times fewer compared to random testing) makes two-stage ST a promising mitigation strategy, if a high enough *r*_*p*_ can be achieved.

### How many antigen tests are needed for pre-screening

In order to determine the most economical number of antigen tests, one can set the condition that these tests need to detect a positive predictive value that is of exactly the right size to achieve the desired reduction in *R*_*eff*_: first, if *r*_*ag*_ is the fraction of the total population that is getting tested by antigen tests, and a fraction *r*_*s*_ of these will get a positive result and therefore constitutes the antigen test positive population to be tested using RNA tests, the fraction of the total population that constitutes the antigen test positive population is *r*_*ag*_*r*_*s*_. Second, if *r*_*mt*_ is the fraction of the population that would need to be RNA tested in a random testing strategy to achieve the desired reduction in *R*_*eff*_ (based on our modeling results [9]), and *r*_*p*_ is the ratio between positive predictive value and the prevalence (see above), the fraction of the population that needs to be tested in a two-stage ST strategy to achieve the same reduction in *R*_*eff*_ is *r*_*mt*_/*r*_*p*_. Third, we set the condition that the population fractions *r*_*ag*_*r*_*s*_ (the antigen test positive population) and *r*_*mt*_/*r*_*p*_ (the population fraction that needs to be tested to achieve the desired reduction in *R*_*eff*_) need to be equal, and resolve for *r*_*ag*_. This leads to the simple expression *r*_*ag*_ = *r*_*mt*_/(*r*_*p*_*r*_*s*_) = *r*_*mt*_/*S*_*e*_.

This implies that the fraction of the overall population to be tested with virus antigen tests every day is *r*_*mt*_/*S*_*e*_ and the fraction of the overall population to which virus RNA tests have to be applied is *r*_*mt*_/*r*_*p*_ = *r*_*mt*_*r*_*s*_/(1 − *f*_*n*_) = *r*_*mt*_(*p* + (1 − *p*)(1 − *S*_*p*_)/*S*_*e*_), with *p* being the overall population prevalence.

**Fig 3:**
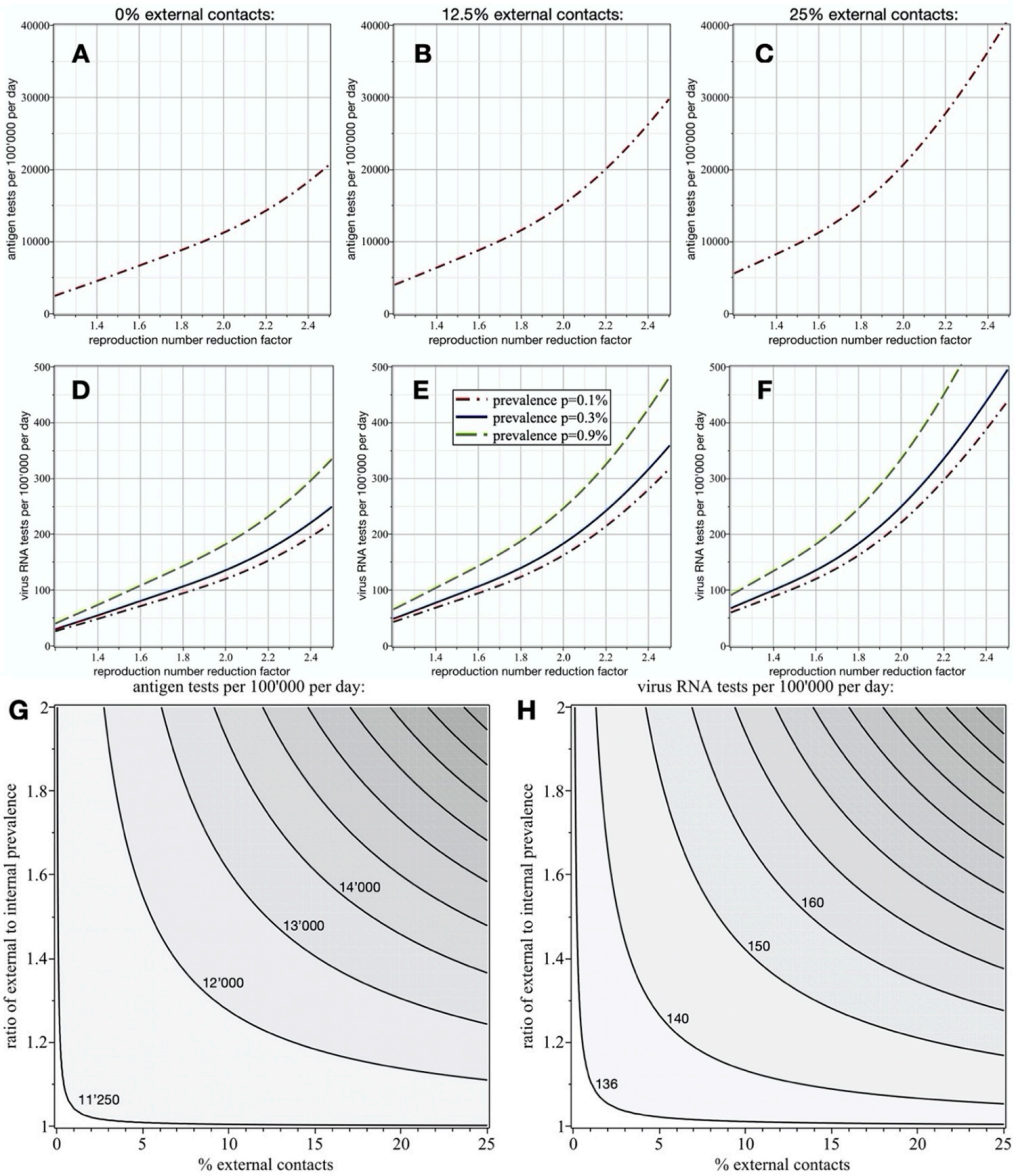
Numbers of required tests per 100’000 people per day as function of reproduction number reduction factor and prevalence p in the undetected population. In the first row (A-C) the number of antigen tests and in the second row (D-F) the number of virus RNA tests are shown. No external contacts are assumed for the results shown in the first column, while 12.5% and 25% external contacts are assumed for the plots in the second and third columns, respectively, where the external population has a two times higher prevalence. As expected, more tests are required to achieve the same reproduction number reduction as the fraction of external infection-relevant contacts increases. Also note that the number of required virus RNA tests increases with a higher overall prevalence (D-F), while the number of antigen tests is independent of p (A-C). Also shown are the effects of fraction of external contacts and ratio of external to internal prevalence on the numbers of required antigen (G) and RNA tests (H) per 100’000 people per day to reduce the reproduction number by a factor of two. A 95% sensitivity and one day delay is assumed for the virus RNA tests, and for the antigen tests sensitivity and specificity of 70% and 99% are assumed.

To give a concrete example, with an overall prevalence of 0.3%, this means that 11’240 antigen tests and 136 virus RNA tests are required per 100’000 people per day to reduce the reproduction number by a factor of two. If the overall prevalence is 0.1% (or 0.9%), then 120 (or 182) virus RNA tests per 100’000 people per day suffice to have the same effect on the reproduction number. The number of required antigen tests, on the other hand, is not affected by the overall prevalence. Assuming respective costs of 57.5CHF and 114.5CHF (current values for Switzerland [13], including all involved personnel charges) for each antigen and RNA test, an average 6.6CHF have to be spent per person per day. This cost is extremely low considering the enormous gain in mitigation and the economic costs of alternative mitigation strategies. Fig 3A and 3D show the numbers of required virus antigen and RNA tests per 100’000 people per day as functions of the reproduction number reduction factor and the overall prevalence *p*. Note that the number of required antigen tests is independent of the overall prevalence (Fig 3A), while more virus RNA tests are required as *p* increases (Fig 3D).

### Two-stage ST limited to sub-populations

So far, our analysis has focused on a closed population. However, a two-stage ST strategy will always be employed on a sub-population (e.g. in a country, state, city, school, or company), which is in constant exchange with other populations. This is especially relevant for specific societal branches, where vaccination is still not available or vulnerables with compromised immunity are prevalent, e.g. primary schools or retirement houses. Next, we therefore investigate scenarios in which two-stage ST is applied within sub-populations only, while no such measures are taken in the remaining population.

Generally, in a focal sub-population in which a fraction *r*_*ec*_ of all infection relevant contacts happens with people external to that sub-population, the virus reproduction number scales with the factor *f*_*ec*_ = (1+ *r*_*ec*_(*r*_*ep*_ − 1)), where *r*_*ep*_ is the ratio of external to internal prevalence. For example, if 25% of the infection relevant contacts are with an external population (*r*_*ec*_ = 0.25), which has a two times higher prevalence (*r*_*ep*_ = 2), then the reproduction number in the sub-population is *f*_*ec*_ = 1.25 times higher than it would be with only internal contacts. This finding allows the quantification of the mitigating effect of two-stage ST, if restricted to a sub-population. To obtain the numbers of required tests (and the resulting costs), one can use the results of two-stage ST in isolated populations (first column in Fig 3) with the modified reproduction number (actual reproduction number without external contacts increased by the factor *f*_*ec*_). Fig 3B, 3C, 3E and 3F show, for a two times higher external prevalence, the respective numbers of required virus antigen and RNA tests per 100’000 people per day as functions of the reproduction number reduction factor and the overall prevalence *p* for *r*_*ec*_ = 12.5% and *r*_*ec*_ = 25%. Fig 3G depicts the number of required antigen and Fig 3H the number of RNA tests per 100’000 people per day to reduce the reproduction number by a factor of two, as functions of fraction of external contacts and ratio of external to internal prevalence. These results suggest that two-stage ST is a viable mitigation strategy, even if applied locally.

### How to deploy two-stage ST

We now have an estimate for the number of antigen and RNA tests needed to achieve a fast and strong reduction in *R*_*eff*_. But once the total number of cases in the population has declined sufficiently, testing can either be reduced or discontinued for a period of time before a new round of tests is initiated. In the following, we study the epidemiological consequences of different deployment strategies of two-stage ST.

**Fig 4:**
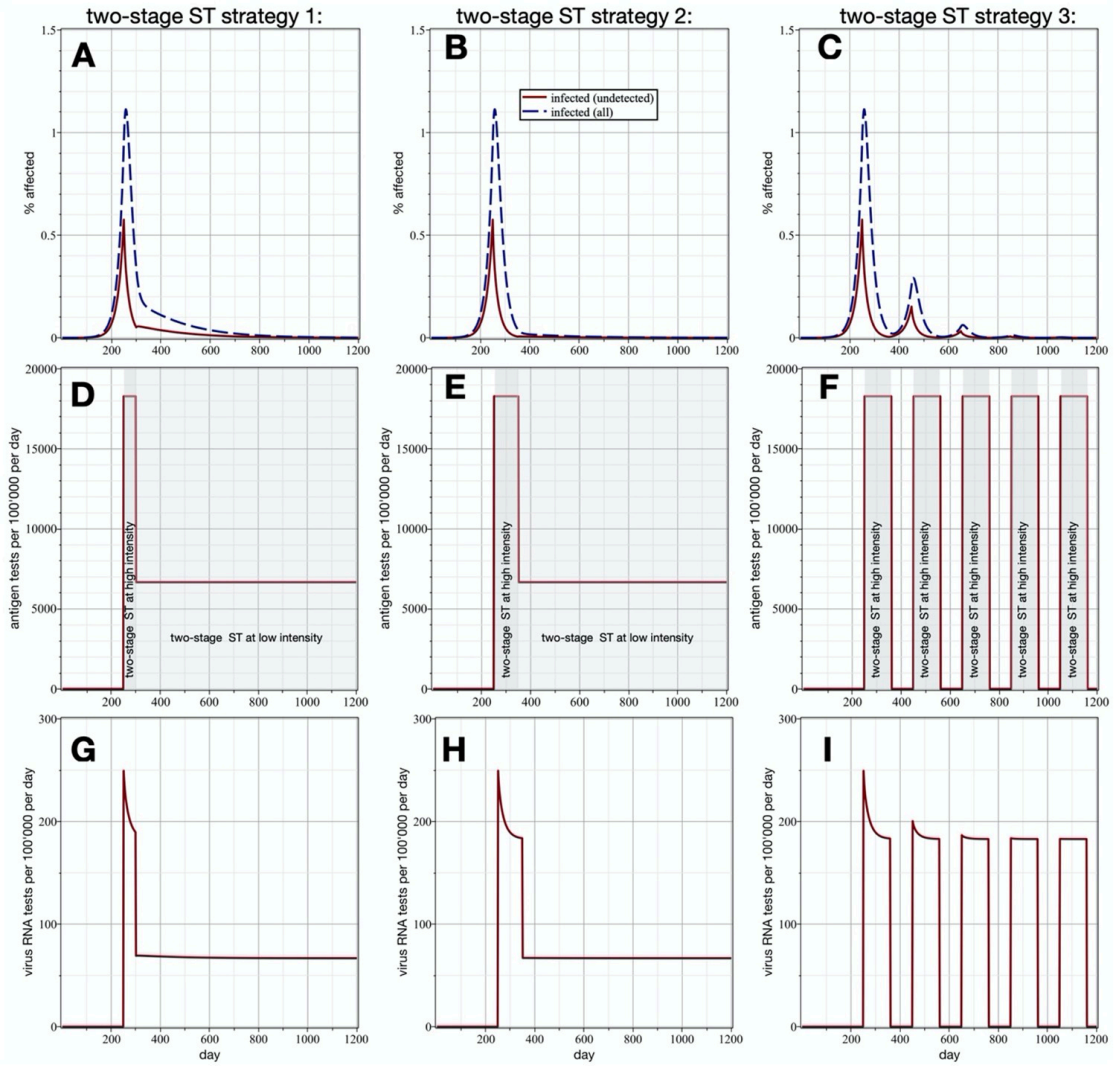
Different two-stage ST mitigation strategies. Shown are overall prevalence and prevalence in the undetected population (A-C; dashed and solid lines, respectively), and number of virus antigen and RNA tests per 100’000 people per day (D-F and G-I, respectively). The unmitigated reproduction number is 1.6 (already reduced by moderate social distancing) and each scenario starts on day 250, when the prevalence just exceeded 1%. The first scenario (first column) follows a two-stage ST strategy, in which for a first period of 50 days 18% of population is virus antigen tested every day. Once the prevalence is reduced by almost one order of magnitude, two-stage ST is continued at a lower intensity, that is, with 7% of the population being antigen tested every day. The second scenario (second column) is identical to the first one, except that the first phase lasts for 100 days, which leads to a reduction of the prevalence by almost two orders of magnitude. In the third scenario (third column), two-stage ST (with 18% of the population being antigen tested every day) is applied in cycles; each cycle starts with 110 days of two-stage ST followed by a 90 day pause. A 95% sensitivity and one day delay is assumed for the virus RNA tests, and for the antigen tests sensitivity and specificity of 70% and 99% are assumed.

Fig 4A–4C show the overall prevalence and prevalence in the undetected population (dashed and solid lines, respectively), and Fig 4D–4F and 4G–4I the number of deployed virus antigen and RNA tests per 100’000 people per day, respectively, as functions of time. The effective reproduction number 1.6 is chosen as a representative example, and it is close to the maximum reproduction numbers observed in the past pandemic waves in Switzerland. Each scenario starts on day 250, when the overall prevalence just exceeded 1%. The first scenario (first column) follows a two-stage ST strategy, in which for a first period of 50 days 18% of the population is virus antigen tested every day. Once the prevalence is reduced by almost one order of magnitude, two-stage ST is continued at a lower intensity, that is, with 7% of the population being antigen tested every day. The uptake of 7%-18% are quite realistic, and in the range of participation rates observed in the mass testing campaign of Canton Grisons [25]. The second scenario (second column) is identical to the first one, except that the first phase lasts for 100 days, which leads to a reduction of the prevalence by almost two orders of magnitude. In the third scenario (third column), two-stage ST (with 18% of the population being antigen tested every day) is applied in cycles; each with 110 days of two-stage ST followed by a pause of 90 days. On the accumulated estimates, we computed the total number of infections, antigen tests and PCR tests for each scenario, per 100’000 people over 1’200 days. The first scenario leads to 5’678 infections, using 6’921’722 antigen tests and 70’961 RNA tests. These estimates become respectively 4’293, 7’502’463 and 76’450 for the second scenario, and 5’418, 10’057’313 and 102’378 for the third one.

## Discussion and conclusion

The two-stage ST approach analyzed here adds to the portfolio of mitigation strategies for the Covid-19 pandemic, and complements approaches like classic contact tracing, hygiene measures, or randomized testing. It could be deployed quickly in countries with sufficient testing capacities like Switzerland (capacity ≈ 230 virus RNA tests per 100’000 per day). Importantly, we show that low sensitivity and specificity of the antigen tests are not inhibitory for the suggested strategy, which allows for faster and easier deployment (e.g. through testing by non-expert personnel, saliva instead of nasal swabs, using less accurate test kits). The earlier such a strategy is adopted, the less logistically and fiscally costly it will be. Our results are in line with Larremore et al. analysis [11] illustrating how a test with much lower molecular sensitivity than PCR can have public health benefits when used frequently.

Two-stage ST, like all testing strategies, has the added benefit of also serving as a surveillance tool, giving decision makers important actionable information on the course of the pandemic. Once two-stage ST is implemented, strategy can be adapted flexibly in response to this information to ensure the desired performance. Monitoring of the prevalence in the population could be performed, for example, by using the relation *p* = (*r*_*s*_ − (1 − *S*_*p*_))/(*S*_*e*_ + *S*_*p*_ − 1), where *r*_*s*_ is the fraction of positive cases when virus antigen testing (with sensitivity and specificity of *S*_*e*_ and *S*_*p*_, respectively) is applied to a representative sub-population.

In the analysis above, we suggest ways to plan an effective two-stage ST campaign, and it is possible to predict its effect on *R*_*eff*_. The required numbers of antigen and virus RNA tests can directly be computed from known quantities (the overall prevalence is not known, but can be estimated based on positive antigen tests). To compensate for statistical noise and modeling uncertainties, we would advise, however, to choose slightly higher test numbers than the calculated ones. Naturally, the mitigation effect of two-stage ST can further be enhanced, if combined with other measures, such as contact tracing, mask wearing, or mild forms of social distancing.

Improved antigen tests with even lower false positive and false negative rates could be used as a stand-alone test for testing random samples of the population. However, developing tests that can detect low virus titers without an amplification step is likely extremely challenging, and the epidemiologically important asymptomatic carriers are most likely to give false negative results. If the currently available low-specificity antigen tests are used as a stand-alone solution to decide whether people should quarantine, this would lead to a large population of healthy people being forced to self-isolate (S6 Fig in S1 File), potentially depressing overall compliance with the strategy. We therefore suggest using tests with significant false-positive rates to be used in two-stage testing schemes such as the one analyzed here.

Currently we are observing a surge in case numbers due to emergence of more infectious variants, while the vaccination is being rolled out world-wide and proved to be significantly effective. However sub-optimal participation in most countries besides waning neutralising antibodies and risk of escape variants, make less intrusive interventions such as repetitive screening, still important; especially for the vulnerable and those age groups who are not covered yet by vaccination programmes. Two-stage ST offers a viable approach to help relax broad social distancing policies without compromising health, while at the same time providing public health officials with much needed actionable information on the success of their interventions. This will be an important prerequisite for reclaiming our normal public life and promoting economic recovery.

## Supporting information

S1 File (ZIP)
