# Supplementary material for: Smart investment of virus RNA testing resources to enhance Covid-19 mitigation
Source: PLoS One. 2021 Nov 30;16(11):e0259018. doi: 10.1371/journal.pone.0259018 (PMC8631684; doi:10.1371/journal.pone.0259018)
Supplement: S1 File — (ZIP) [file pone.0259018.s001.zip › S1_File.pdf]

Smart investment of virus RNA testing resources to enhance Covid-19 mitigation

Hossein Gorji[1¶*], Markus Arnoldini[2¶], David F. Jenny[3], Wolf-Dietrich Hardt[4 &], Patrick Jenny[5 &]

1 Laboratory of Multiscale Studies in Building Physics, Empa, Dübendorf, Switzerland
2 Department of Health Sciences and Technology, Swiss Federal Institute of Technology, CH-8093 Zürich, Switzerland
3 Department of Mathematics, Swiss Federal Institute of Technology, CH-8093 Zürich, Switzerland
4 Institute of Microbiology, D-BIOL, Swiss Federal Institute of Technology, CH-8093 Zürich, Switzerland
5 Department of Mechanical and Process Engineering, Swiss Federal Institute of Technology, IFD, CH-8092 Zürich, Switzerland

¶These authors contributed equally to this work.
& These authors also contributed equally to this work.
* mohammadhossein.gorji@empa.ch

Supporting information

Based on Eq. (22) we can estimate the number of required tests per day for a given reduction in the reproduction number (reproduction number reduction factor), i.e., $\mathcal{R}_{\text{eff}}^{wt}/\mathcal{R}_0$, as a function of test characteristics and cross-infections. We consider a RNA mass-testing scenario for different test processing times, i.e., for $\tau_{proc} \in \{0.5, 1, 1.5\}$ days, different participation fractions, i.e., for $t \in \{0.6, 0.8, 1\}$, and different sensitivity, i.e., for $S_e \in \{0.95, 0.85\}$. While it is observed that sensitivity and specificity of RNA tests depend on the time passed from infection [26], here for simplicity we consider a constant sensitivity among all tested compartments. In particular a lower sensitivity within the exposed population reduces the effect of repetitive testing on the reproduction number. Furthermore the cross infections are characterized by the fraction of external contacts $r_{ec} \in \{0\%, 12.5\%, 25\%\}$, where the prevalence ratio of external vs. internal population is assumed to be $p^e/p = 2$. We applied a Monte-Carlo technique to compute the detection rates. The results for the given parameter set are shown in S1 Fig(a) and (b) for 100% participation without external infections for $S_e = 95\%$ and $S_e = 85\%$, respectively, in S2 Fig(a) and (b) with without external infections and $S_e = 95\%$ for $t = 60\%$ and $t = 80\%$, respectively, and in S3 Fig(a) and (b) for 100% participation and $S_e = 95\%$ for 12.5% and 25% external contacts, respectively.

S1 Fig. Mass-testing: A mitigation strategy relying on RNA mass-testing with 100% participation is assumed and we computed the number of tests performed per day, which are needed to achieve a particular test-speed dependent $\mathcal{R}_{\text{eff}}^{wt}/\mathcal{R}_0$ ratio; for (a) 95% and for (b) 85% sensitivity. Test speeds were: 0.5 days (black line), 1 day (orange line) and 1.5 days (blue line). The green dashed line indicates reduction of reproduction number from 1.6 to $\mathcal{R}_{\text{eff}}^{wt} = 1$ via mass-testing.

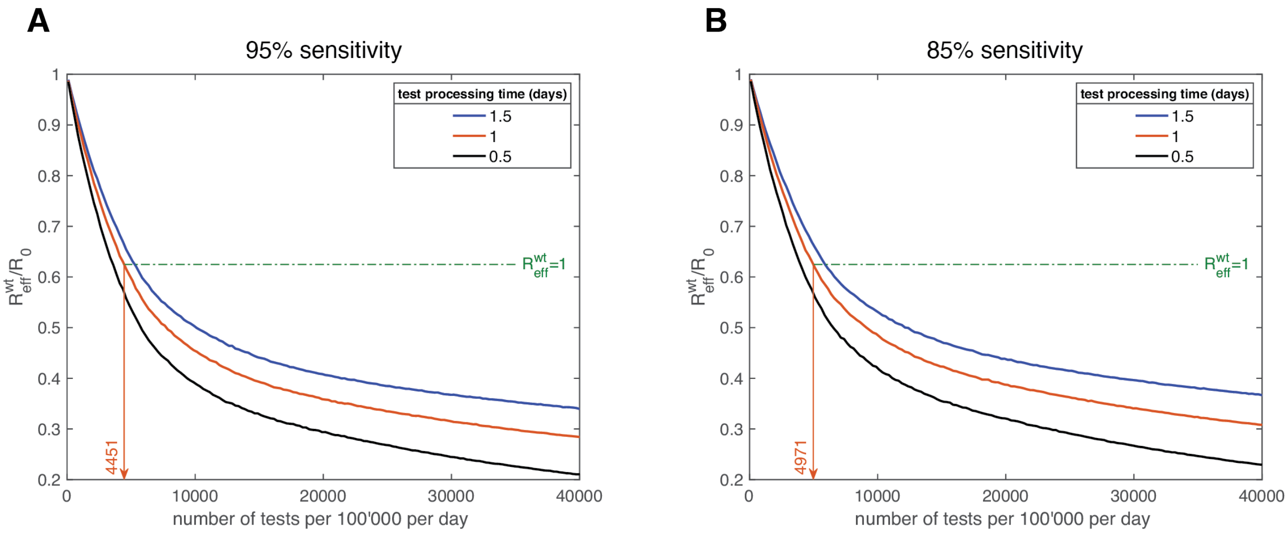

S2 Fig. Mass-testing: A mitigation strategy relying on RNA mass-testing with 95% sensitivity is assumed and we computed the number of tests performed per day, which are needed to achieve a particular test-speed dependent $\mathcal{R}_{\text{eff}}^{wt}/\mathcal{R}_0$ ratio; for (a) 60% and (b) 80% participation. Test speeds were: 0.5 days (black line), 1 day (orange line) and 1.5 days (blue line). The green dashed line indicates reduction of reproduction number from 1.6 to $\mathcal{R}_{\text{eff}}^{wt} = 1$ via mass-testing.

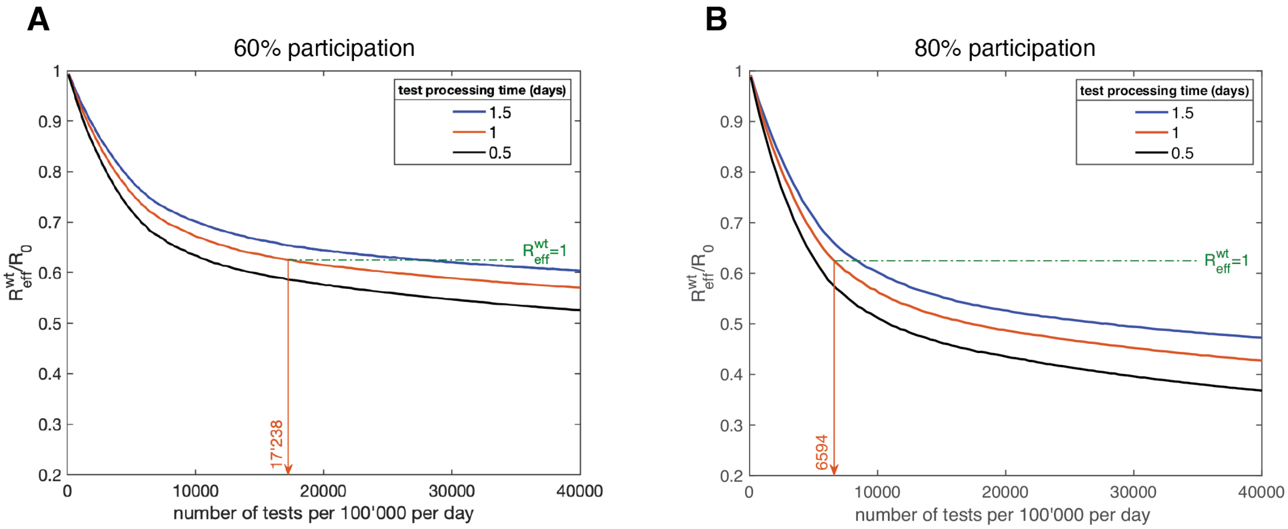

S3 Fig. Mass-testing: A mitigation strategy relying on RNA mass-testing with 100% participation within the considered population and with 95% sensitivity is assumed and we computed the number of tests performed per day, which are needed to achieve a particular test-speed dependent $\mathcal{R}_{\text{eff}}^{wt}/\mathcal{R}_0$ ratio; for (a) 12.5% and for (b) 25% external contacts. A prevalence ratio of 2 between the external and internal populations is considered. Test speeds were: 0.5 days (black line), 1 day (orange line) and 1.5 days (blue line). The green dashed line indicates reduction of reproduction number from 1.6 to $\mathcal{R}_{\text{eff}}^{wt} = 1$ via mass-testing.

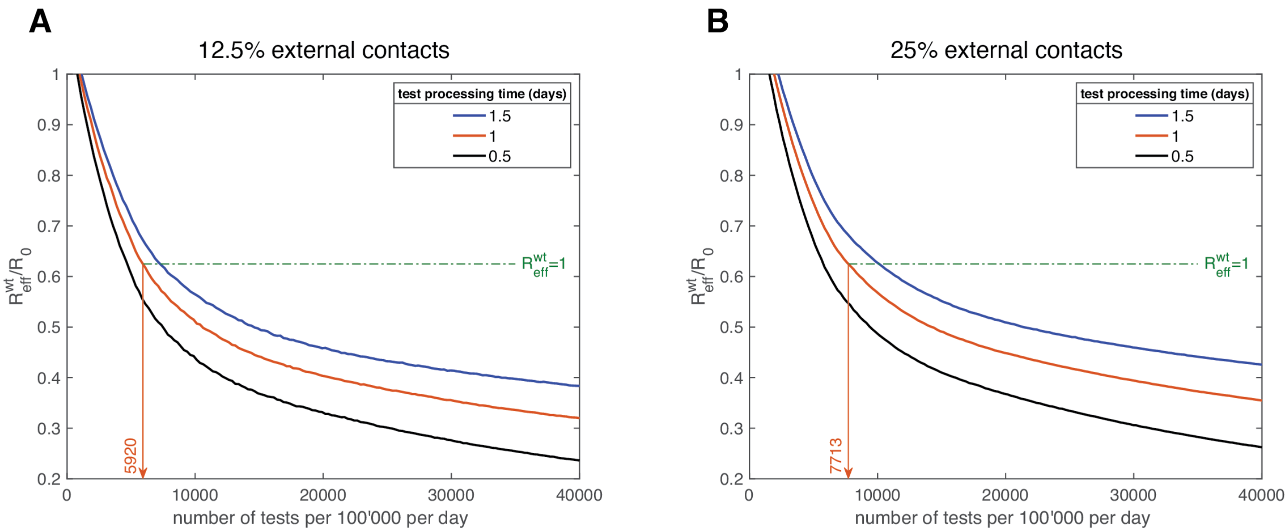

To illustrate the performance of the two-stage testing mitigation, let us consider a concrete example. Suppose that the available antigen tests have sensitivity and specificity of $S_e = 70\%$ and $S_p = 99\%$, respectively. Furthermore, suppose that the pandemic is at an early stage with $p^l = 0.3\%$. The RNA tests are assumed to have sensitivity of 95% and the total processing time is considered is $\tau_{\text{proc}} \in \{0.5, 1, 1.5\}$ days. Further, 100% participation within the considered population is assumed, but we account for cross-infections via contact with an external population, which has a two times higher prevalence, where the ratio of external to overall contacts is $r_{ex} \in \{0, 0, 12.5, 25\}$. S4 Fig (a) & (b) show results for the required numbers of antigen and RNA tests, respectively, for the case without cross-infection. The number of required RNA tests is reduced by the factor 58 compared to the simple mass-testing strategy. Furthermore, S5 Fig (a) & (b) show the respective numbers of required antigen and RNA tests for 12.5% external contacts, and S5 Fig (c) & (d) show the same data for 25% external contacts. As expected, more mixing with higher prevalence external population puts more burden on the numbers of tests. Nevertheless, the numbers of required antigen and RNA tests still are in the range of available resources. S6 Fig shows the fraction of infected cases among all people with positive virus antigen test result as function of prevalence; the left plot for 85%, 70% and 55% sensitivity and a fixed specificity of 99%; the right plot for 99.5%, 99% and 98.5% specificity and a fixed sensitivity of 70%. The fraction of actually infected cases among positively tested individuals can be calculated as $pS_e/(pS_e + (1-p)(1-S_p))$.

S4 Fig. Two-stage testing: A mitigation strategy relying on pre-screening using mass antigen testing and then RNA testing on the positive cases is assumed, and we computed the number of tests performed per day (assuming 100% participation), which are needed to achieve a particular test-speed dependent $\mathcal{R}^{wt}_{\text{eff}}/\mathcal{R}_0$ ratio at 95% sensitivity of RNA test results, 70% and 99% sensitivity and specificity of antigen test results, respectively, and an overall prevalence of 0.3%. Combined test-to-quarantine speeds are 0.5 days (black line), 1 day (orange line) and 1.5 days (blue line).

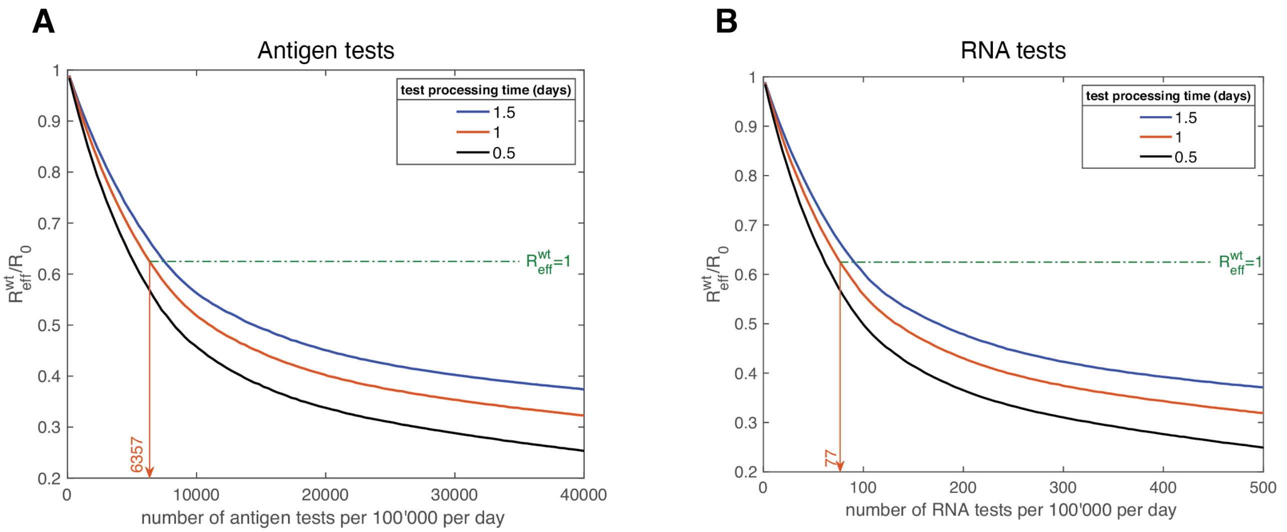

S5 Fig. Two-stage testing: A mitigation strategy relying on pre-screening using mass antigen testing and then RNA testing on the positive cases is assumed, and we computed the number of tests performed per day (assuming 100% participation), which are needed to achieve a particular test-speed dependent $\mathcal{R}_{\text{eff}}^{wt}/\mathcal{R}_0$ ratio at 95% sensitivity of RNA test results, 70% and 99% sensitivity and specificity of antigen test results, respectively, and overall an prevalence of 0.3%; for (a) and (b) with 12.5% external contacts; for (c) and (d) with 25% external contacts. A prevalence ratio of 2 between the external and internal populations is assumed. Combined test-to-quarantine speeds were 0.5 days (black line), 1 day (orange line) and 1.5 days (blue line).

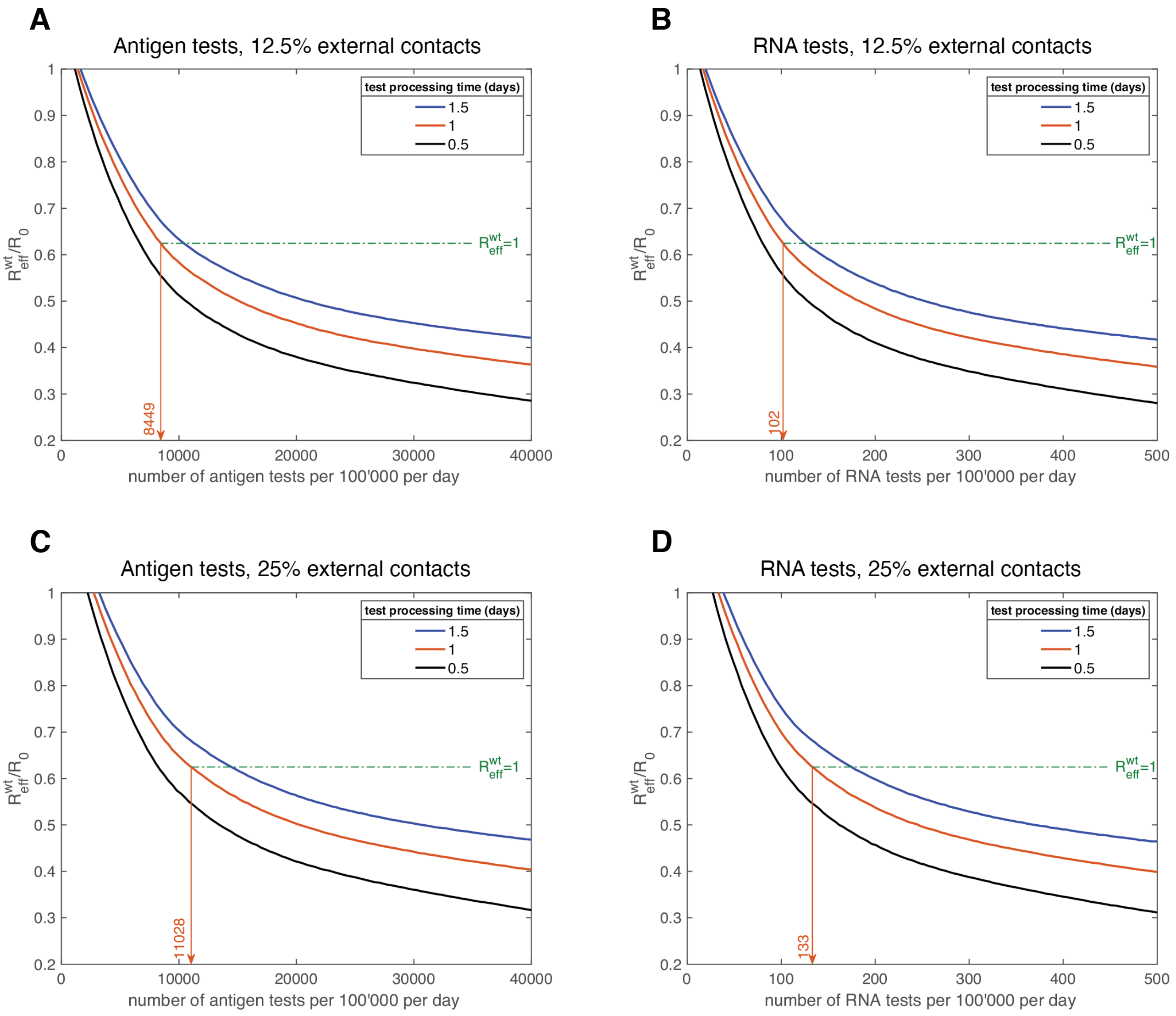

S6 Fig. Fraction of infected cases among all people with positive virus antigen test result as function of prevalence. Left: for 85%, 70% and 55% sensitivity and a fixed specificity of 99%; right: for 99.5%, 99% and 98.5% specificity and a fixed sensitivity of 70%. The fraction of actually infected cases among positively tested individuals can be calculated as $pS_e/(pS_e + (1-p)(1-S_p))$.

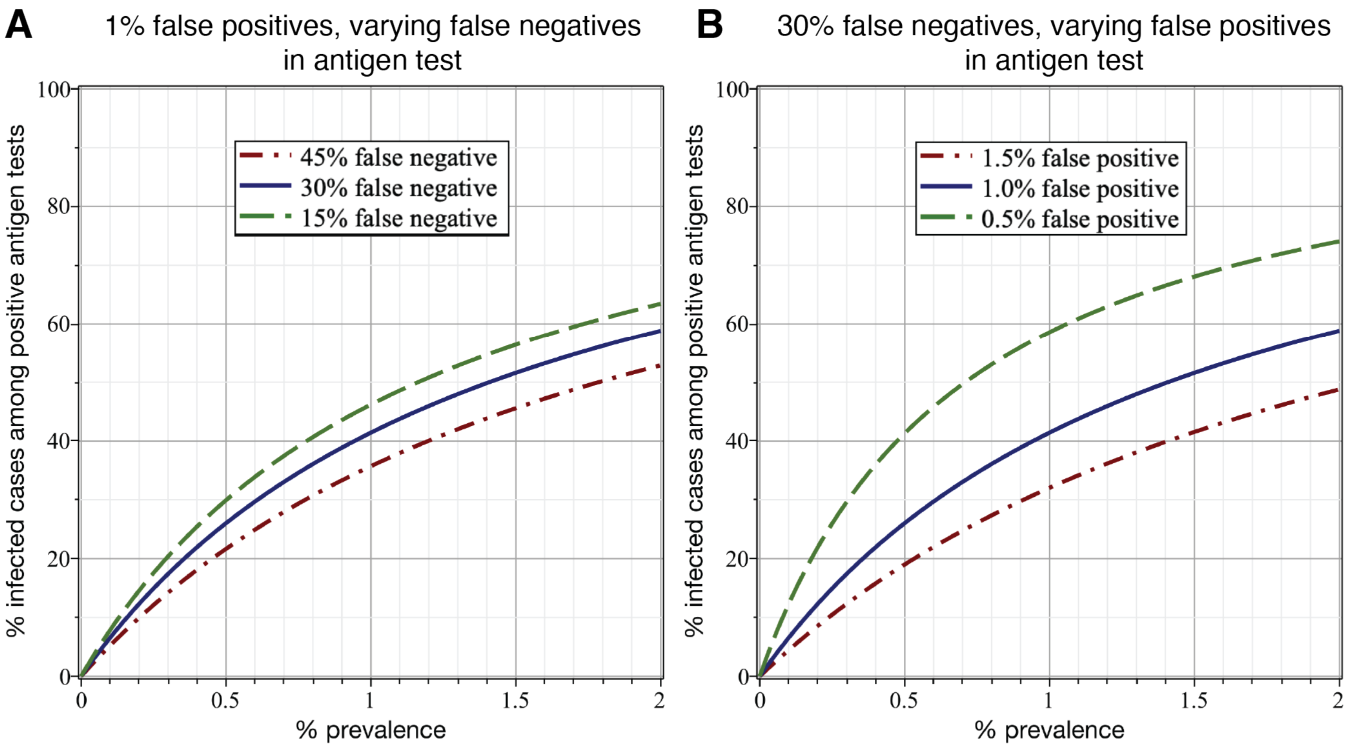

Model Implementation

The calculations for mass-testing and two-stage testing were implemented with MATLAB and the Statistics Toolbox Release 2018b. The corresponding codes are available on GitHub server via `https://github.com/gorjih2/STeCC_preliminary`.

Acknowledgments

Hossein Gorji acknowledges the funding provided by Swiss National Science Foundation under the grant number 174060. The funders had no role in study design, data collection and analysis, decision to publish, or preparation of the manuscript.

References

1. Bongiorno C, Cagnol J. Modeling the emergence of vaccine-resistant variants with Gaussian convolution COVID-19: Could the wrong strategy ruin vaccine efficiency? MedRxiv. 2021.

2. Li R, Pei S, Chen B, Song Y, Zhang T, Yang W, Shaman J. Substantial undocumented infection facilitates the rapid dissemination of novel coronavirus (SARS-CoV2) Science. 2020

3. Siettos CI, Russo L. Mathematical modeling of infectious disease dynamics. Virulence. 2013 4 (4):295–306.

4. Ferguson N, Laydon D, Nedjati-Gilani G, Imai N, Ainslie K, Baguelin M, Bhatia S, Boonyasiri A, Cucunubá Z, Cuomo-Dannenburg G et al. Impact of

non-pharmaceutical interventions (NPIs) to reduce COVID-19 mortality and healthcare demand London: Imperial College COVID-19 Response Team, March. 2020 16.

5. Nishiura H, Kobayashi T, Miyama T, Suzuki A, Jung SM, Hayashi K, Kinoshita R, Yang Y, Yuan B, Akhmetzhanov AR et al. Estimation of the asymptomatic ratio of novel coronavirus infections (COVID-19) International journal of infectious diseases. 2020 94 (154).

6. Kimball A, Hatfield KM, Arons M, James A, Taylor J, Spicer K, Bardossy AC, Oakley LP, Tanwar S, Chisty Z et al. Asymptomatic and presymptomatic SARS-CoV-2 infections in residents of a long-term care skilled nursing facility—King County, Washington, March 2020 Morbidity and Mortality Weekly Report. 2020 69 (13).

7. Ferretti L, Wymant C, Kendall M, Zhao L, Nurtay A, Abeler-Dörner L, Parker M, Bonsall D, Fraser C. Quantifying SARS-CoV-2 transmission suggests epidemic control with digital contact tracing Science. 2020.

8. Lavezzo E, Franchin E, Ciavarella C, Cuomo-Dannenburg G, Barzon L, Del Vecchio C, Rossi L, Manganelli R, Loregian A, Navarin N et al. Suppression of COVID-19 outbreak in the municipality of Vo, Italy MedRxiv. 2020.

9. Gorji H, Arnoldini M, Jenny D, Hardt WD, Jenny P. Dynamic modelling to identify mitigation strategies for the COVID-19 pandemic Swiss Medical Weekly. 2021 151.

10. Grassly NC, Pons-Salort M, Parker EPK, White PJ, Ferguson NM, Ainslie K, Baguelin M, Bhatt S, Boonyasiri A, Brazeau N et al. Comparison of molecular testing strategies for COVID-19 control: a mathematical modelling study The Lancet Infectious Diseases. 2020 20 (12), 1381–1389.

11. Larremore DB, Wilder B, Lester E, Shehata S, Burke JM, Hay JA, Tambe M, Mina MJ, Parker R. Test sensitivity is secondary to frequency and turnaround time for COVID-19 screening Science advances. 2021 7 (1).

12. Scire J, Nadeau S, Vaughan T, Brupbacher G, Fuchs S, Sommer J, Koch KN, Misteli R, Mundorff L, Götz T, Eichenberger T et al. Reproductive number of the COVID-19 epidemic in Switzerland with a focus on the Cantons of Basel-Stadt and Basel-Landschaft Swiss Medical Weekly. 2020, 1424-3997.

13. Robert F. Fast, cheap tests could enable safer reopening Science. 2020, 608–609.

14. Wu JT, Leung K, Leung GM. Nowcasting and forecasting the potential domestic and international spread of the 2019-nCoV outbreak originating in Wuhan, China: a modelling study The Lancet. 2020 395, 689–697.

15. Hou C, Chen J, Zhou Y, Hua L, Yuan J, He S, Guo Y, Zhang S, Jia Q, Zhao C et al. The effectiveness of quarantine of Wuhan city against the Corona Virus Disease 2019 (COVID-19): A well-mixed SEIR model analysis Journal of medical virology. 2020.

16. Radulescu A, Cavanagh K. Management strategies in a SEIR model of COVID 19 community spread arXiv preprint arXiv:2003.11150. 2020

17. He S, Peng Y, Sun K. SEIR modeling of the COVID-19 and its dynamics Nonlinear Dynamics. 2020, 1-14.

18. Brauer F. Compartmental models in epidemiology Mathematical epidemiology, Springer, 2008.

19. Furukawa NW, Brooks JT, Sobel J. Evidence supporting transmission of severe acute respiratory syndrome coronavirus 2 while presymptomatic or asymptomatic Emerging infectious diseases. 2020 26 (7).

20. Savvides C, Siegel R. Asymptomatic and presymptomatic transmission of SARS-CoV-2: A systematic review MedRxiv. 2020.

21. Dansu EJ, Seno H. A model for epidemic dynamics in a community with visitor subpopulation Journal of theoretical biology. 2019 478, 115-127.

22. Gorji H, Arnoldini M, Jenny DF, Hardt WD, Jenny P. STeCC: Smart Testing with Contact Counting Enhances Covid-19 Mitigation by Bluetooth App Based Contact Tracing MedRxiv. 2020

23. Sheridan C. Fast, portable tests come online to curb coronavirus pandemic Nat Biotechnol. 2020 10.

24. Zhou F, Yu T, Du R, Fan G, Liu Y, Liu Z, Xiang J, Wang Y, Song B, Gu X et al. Clinical course and risk factors for mortality of adult inpatients with COVID-19 in Wuhan, China: a retrospective cohort study The Lancet. 2020.

25. Gorji H, Lunati I, Rudolf F, Vidondo B, Hardt WD, Jenny P, Engel D, Schneider J, Jamnicki M, Leuthold R et al. Results from Canton Grisons of Switzerland Suggest Repetitive Testing Reduces SARS-CoV-2 Incidence (February-March 2021) MedRxiv. 2021.

26. Böger B, Fachi MM, Vilhena RO, de Fátima Cobre A, Tonin FS, Pontarolo R. Systematic review with meta-analysis of the accuracy of diagnostic tests for COVID-19 American journal of infection control. 2020.
